# Construction of *Escherichia coli* Mutant with Decreased Endotoxic Activity by Modifying Lipid A Structure

**DOI:** 10.3390/md13063388

**Published:** 2015-05-27

**Authors:** Qiong Liu, Yanyan Li, Xinxin Zhao, Xue Yang, Qing Liu, Qingke Kong

**Affiliations:** 1 Institute of Preventive Veterinary Medicine, College of Veterinary Medicine, Sichuan Agricultural University, Chengdu 611130, China; E-Mails: p19890528@126.com (Q.L.); xxinzhao@163.com (X.Z.); yx1207@foxmail.com (X.Y.); 2 State Key Laboratory of Food Science and Technology, Jiangnan University, Wuxi 214122, China; E-Mail: yanyanli@jiangnan.edu.cn; 3 Department of Bioengineering, College of Veterinary Medicine, Sichuan Agricultural University, Chengdu 611130, China

**Keywords:** *Escherichia coli* BL21 (DE3), lipid A, *pagL*, *lpxE*, protein expression

## Abstract

*Escherichia coli* BL21 (DE3) and its derivatives are widely used for the production of recombinant proteins, but these purified proteins are always contaminated with lipopolysaccharide (LPS). LPS is recognized by the toll-like receptor 4 and myeloid differentiation factor 2 complex of mammalian immune cells and leads to release of pro-inflammatory cytokines. It is a vital step to remove LPS from the proteins before use for therapeutic purpose. In this study, we constructed BL21 (DE3) ∆*msbB28* ∆*pagP38* mutant, which produces a penta-acylated LPS with reduced endotoxicity. The plasmids harboring *pagL* and/or *lpxE* were then introduced into this mutant to further modify the LPS. The new strain (S004) carrying plasmid pQK004 (*pagL* and *lpxE*) produced mono-phosphoryated tetra-acylated lipid A, which induces markedly less production of tumor necrosis factor-α in the RAW264.7 and IL-12 in the THP1, but still retains ability to produce recombinant proteins. This study provides a strategy to decrease endotoxic activity of recombinant proteins purified from *E*. *coli* BL21 backgrounds and a feasible approach to modify lipid A structure for alternative purposes such as mono-phosphoryl lipid A (MPL) as vaccine adjuvants.

## 1. Introduction

*Escherichia coli* BL21 (DE3) and its derivatives are widely used for recombinant protein expression due to its high transformation efficiency, large recombinant protein yield and ease of use [1,2]. However, lipopolysaccharide (LPS, also called endotoxin) is co-purified with recombinant proteins from this line of *E*. *coli* strains, intrinsically limiting their use in humans and other mammals [3,4]. LPS must therefore be removed before BL21-generated recombinant proteins are used for therapeutic purposes or raising antibodies [5]. LPS is a predominant structural component on the cell envelope of Gram-negative bacteria, coating approximately three-fourths of the outer membrane. LPS has three different regions: a hydrophobic lipid A, a short non-repeating core oligosaccharide (C-OS), and a distal polysaccharide termed O-antigen (O-PS) which is the endmost distal to the outer membrane [1,6,7]. Lipid A is the component of LPS that can activate both the TLR4/MD-2/CD14 pathway and caspase 11 pathway [8] resulting in an immuno-inflammatory response. Overstimulation of innate immunity by lipid A will lead to organ damage, severe shock, even death of mammals and humans [9,10]. As lipid A is a minimum structural component necessary for *E. coli* BL21 growth in the laboratory condition [11], we cannot remove lipid A structure from *E*. *coli* BL21 to avoid LPS contamination during protein purification; however, we are able to modify lipid A structure to decrease or eliminate its endotoxic property by genetic engineering approaches [8].

*E. coli* lipid A contains two phosphate groups and six acyl chains, which is the most efficient form for activating pro-inflammatory responses [6]. Several proteins are involved in altering the number of acyl chains in *E. coli* lipid A, including HtrB (LpxL), MsbB (LpxM), PagP, PagL and LpxR. LpxL and LpxM are responsible for the addition of acyl chains in lipid A synthesis, and PagP controls the late decoration of acyl chains. LpxR and PagL will catalyze removal of an acyl chain from lipid A [12]. *msbB* encodes a ligase responsible for addition of the myristic acid moiety to the lipid A in the last step of lipid A synthesis, though not essential for bacterial growth [13], as the ∆*msbB* mutant grows normally and produces a non-myristylated LPS [12]. The lipid A from *E. coli msbB* mutant lacking myristoyl fatty acid moiety could reduce the ability to stimulate immune cells to produce E-selectin and TNF-α [14,15,16]; for example, ∆*msbB* derivatives have been used to develop live vaccines to treat cancer [17]. PagP catalyzes addition of a phospholipid-derived palmitate chain to the hydroxyl of the *R*-3′-hydroxymyristate chain at the 2 position of lipid A [18,19]. A *pagP* gene is present in the genome of *E*. *coli* BL21 (DE3), which is not active when grown under normal conditions. Palmitoylation of lipid A by PagP will lead to an increased resistance to cationic antimicrobial peptides (CAMPs) in *E*. *coli* [19,20,21]. Although it was not discovered in *E. coli*, PagL derived from *S. typhimurium* was shown to hydrolyze the ester bond at the position of lipid A and also was able to function in *E. coli*, thereby releasing the primary 3-hydroxymyristoyl moiety [22,23]. Deacylation by PagL modulates the recognition of lipid A by the TLR4/MD-2 complex and decreases its ability to induce pro-inflammatory response [24,25]. LpxE, an inner membrane phosphatase from *Francisella tularensis* subspecies *novicida* can selectively remove the 1′-phosphate group of lipid A in *E. coli* and *Salmonella* [26,27], generating a structure similar to mono-phosphoryl lipid A (MPL) that remains covalently linked to LPS.

The total number, length and position of the acyl chains and two phosphate groups at the 1 and 4′ positions are critical factors for full lipid A activation of TLR4/MD2 and caspase-11 pathway [28,29,30,31]. One strategy to decrease the endotoxic activity is to reduce the number of fatty acyl chain and/or remove phosphate group from lipid A structure [32]. In this study, we aim at maximally reducing lipid A endotoxicity by altering the number of fatty acid chains and removing 1′-phosphate group of lipid A via deleting *msbB* and *pagP* and overexpressing *lpxE* and *pagL* in the BL21 (DE3) strain for the purpose of expressing proteins with low endotoxic activity.

## 2. Materials and Methods

### 2.1. Bacterial Strains, Plasmids, Media, and Growth Conditions

*Escherichia coli BL21 (DE3)* (NEB) and its derivatives were routinely grown at 37 °C in lysogeny broth (LB) [33] or on LB agar. When required, antibiotics were added at an appropriate final concentration (μg/mL), 50 for kanamycin (Kan), 25 for chloramphenicol (Cm). Diaminopimelic acid (DAP) was added to a concentration of 50 μg/mL for the growth of *E. coli* strain χ7213 [34]. LB agar containing 5% sucrose was used for *sacB* gene-based counter-selection in allelic-exchange experiments.

### 2.2. Construction of Plasmids and Bacterial Strains

DNA manipulations were carried out as described [35]. Transformation of *E. coli* was performed by electroporation. Transformants were selected on LB agar plates containing appropriate antibiotics. The primers used in this study are listed in Table 1. For construction of the ∆*msbB* mutation, which deleted the entire *msbB* open reading frame, the BL21 (DE3) genome was used as a template for PCR. Fragments in the amount of 250-bp DNA containing the region upstream of the *msbB* gene and downstream of the *msbB* gene using primers msbB-1F/1R and msbB-2F/2R were amplified, respectively (Table 1). The two PCR fragments were purified in agarose gels, combined at a 1:1 molar ratio, and joined by PCR using primers msbB-1F and msbB-2R. The resulting PCR product with A-terminal was inserted to T-terminal pRE112 vector generated by *Ahd*I digestion [36], resulting in plasmid pQK001. The same strategy was used to construct pQK002 for *pagP* deletion in BL21 (DE3) (Table 2).

The plasmid pYA4291 containing a fragment of ∆*lpxR*:P_lpp_ *pagL* was used as a template for constructing a plasmid to express *pagL*. Briefly, the fragment of lpxRU-P_lpp_ *pagL*-lpxRD was amplified using primers lpxRscreenF and lpxRscreenR, and ligated to a T-terminal vector p15a generated by *Ahd*I digestion, resulting in plasmid pQK003. Using the same strategy, pQK004 was constructed to express *lpxE* based on pYA4295 [37]. pQK005 was constructed by inserting the fragment of lpxRU-P_lpp_ *pagL*-lpxRD to blunt plasmid pQK004 generated by *Sna*BI digestion (Table 2).

A plasmid pQK006 was constructed for protein expression in BL21. A *phoP* gene was amplified from the genome of *Pasteurella multocida* using the primer pairs phoP-F/phoP-R which contained restriction sites *Nde*I and *Bam*HI*.* The PCR product was digested and ligated to the same enzyme-digested vector pET-32a, resulting in pQK005. All the plasmids in this study were sequenced to confirm their correct insertion.

**Table 1:** Primers used in this study.

Primers	Sequences (5′–3′)	Function
msbB-1F	CAGTTCGACAATGTGGAAGAAG	For deletion of *msbB* by suicide plasmid
msbB-1R	ACCTGCAGGATGCGGCCGCGG GCCTCTCGCGAGG	
msbB-2F	CCGCGGCCGCATCCTGCAGGT GCTTTTCCAGTTTCGG	
msbB-2R	GCGTTATATGCACTTGCGC	
pagP-1F	CCTTGATTGCATTTTGTCAT	For deletion of *pagP* by suicide plasmid
pagP-1R	GTCTCACCCGGGCCTGCAGGTTGTGACCATAAAACATTTA	
pagP-2F	CACAACCTGCAGGCCCGGGTGAGACAAATGAAGTTTTAGT	
pagP-2R	TGCTGCCGTCTTCCGGAGTA	
pagPscreenF	AAACGCCGTTAACCCGATA	Insert P_lpp_ *lpxE* to p15a vector
pagPscreenR	TAGACACAAATGCTGCTGTGTCG	
lpxRscreenF	CAGGGGGTGTCAGTATTTGGCG	Insert P_lpp_ *pagL* to p15a vector
lpxRscreenR	CGTGAAGCCAATAATTTCTCGCAC	
phoP-F	TCATATGCGAATTTTATTAATAGAATATG	Insert *phoP* gene to pET-32a for expression phoP protein from *Pasteurella*
phoP-R	TGGATCCTTAAGCCATTTCATCATTTTTTCC	

**Table 2:** Bacterial strains and plasmids used in this study.

Strains or Plasmids	Description	Source
Strains		
*E. coli* BL21 (DE3)	Expression protein strain	NEB
S001	BL21 (DE3) ∆*msbB28* ∆*pagP38*	This work
S002	S001 with pQK003	This work
S003	S001 with pQK004	This work
S004	S001 with pQK005	This work
χ7232	*endA1 hsdR17* (*rK−*, *mK+*) *supE44 thi-1 recA1 gyrArelA1Δ* (*lacZYA-argF*) *U169*λpir*deoR* (φ*80dlac Δ* (*lacZ* ) *M15*)	[37]
χ7213	thi-1 thr-1 leuB6 glnV44 tonA21 lacY1 recA1 RP4-2-Tc:μλpir Δ*asdA4* Δ*zhf-2*:Tn 10	[37]
Plasmids		
p15a T-vector	Low copy expression plasmid	[38]
pET-32a	Protein expression	Novagen
pQK001	For deletion of *msbB* in BL21	This work
pQK002	For deletion of *pagP* in BL21	This work
pYA4291	P_lpp_ *pagL* in pRE112	Lab collection
pYA4295	P_lpp_ *lpxE* (codon-optimized) in pRE112	[39]
pQK003	Insert P_lpp_ *pagL* to p15a and express PagL for deacylation of lipid A	This work
pQK004	Insert P_lpp_ *lpxE* to p15a and express *lpxE* to remove the 1-phosphate group of lipid A	This work
pQK005	Insert P_lpp_ *pagL* and P_lpp_ *lpxE* to p15a Express both *pagL* and *lpxE* to modify the lipid A	This work
pQK006	Expression of PhoP protein of *Pasteurella multocida*	Lab collection

### 2.3. BL21 (DE3) Mutant Strain Construction

∆*msbB* and ∆*pagP* mutation in the BL21 (DE3) were constructed by suicide plasmids method [40]. Briefly, the bacterial cultures of the BL21 (DE3) and the *E*. *coli* χ7213 harboring suicide plasmids pQK001 (for ∆*msbB*) or pQK002 (for ∆*pagP*) were conjugated at the ratio of 1:2 on LB solid agar plate overnight, and then the mixed bacteria were streaked on LB plates containing 25 μg/mL Cm and incubated overnight. Isolated colonies, in which the suicide plasmid was integrated into the BL21 genome, were grown on LB Cm plates. Cm-resistant colonies were propagated in LB liquid media 4 h, diluted, and spread on LB plates containing 5% sucrose. Colonies which have a sucrose-resistant and Cm-sensitive phenotype were screened by colony PCR. Using the same strategy, the second mutation ∆*pagP* could be introduced into the ∆*msbB* mutant to generate double mutant S001.

### 2.4. Analysis of Characteristics of BL21 (DE3) Mutant Strains

Ten microliters of BL21 (DE3) and mutant strains were inoculated into a tube containing 2 mL of LB liquid medium and incubated overnight at 37 °C. Of these cultures, 1 mL was subsequently grown in 99 mL LB liquid medium and growth was estimated at 1 h intervals by measuring the optical density (OD_600_) of the culture using the Spectronic 20. Growth curves were measured in triplicate using three biological replicates.

Plasmid pQK006 was introduced into competent BL21 (DE3) and modified progeny strains, and transformants were grown to an OD_600_ of 0.6 at 37 °C in LB medium supplemented with 50 μg/mL kanamycin and 25 μg/mL Cm when needed. Expression of recombinant protein was induced by addition of isopropyl β-d-1-thiogalactopyranoside (IPTG) (final concentration, 1 mM) for 4 h. Bacteria were harvested and lysed by sonication in 25 mM Tris-HCl pH 8.0, 1.15 mM EDTA, 1 mg/mL lysozyme. Cell lysates were centrifuged at 12,000× *g* for 5 min. The supernatant contained the soluble fraction while the pellets were the insoluble material. Fractions were suspended in SDS-PAGE loading buffer (50 mM Tris-HCl pH 6.8, 0.1 M dithiothreitol, 2% SDS, 0.1% bromophenol blue, 10% glycerol). The different fractions were separated by SDS-PAGE and stained by Coomassie blue.

### 2.5. Lipid A Isolation and Mass Spectrometry Procedures

Lipid A isolation was performed as described previously with some modifications [27]. Briefly, bacteria in 200 mL cultures were grown to an OD_600_ of 1.0. The cells were pelleted and washed with PBS twice. The cell pellet was resuspended in a total volume of 76 mL single-phase Bligh-Dyer mixture containing chloroform, methanol and water (1:2:0.8 v/v), mixed and agitated for 1 h at room temperature. The cell fragments were harvested by centrifugation at 2000 rpm for 20 min, and the pellets were washed twice using the same solution to remove the soluble material. A total of 25 mL 12.5 mM sodium acetate (pH 4.5) was added to suspend the pellets, which were sonicated and then heated to 100 °C for 30 min for releasing lipid A from LPS. After cooling to room temperature, the suspensions were re-extracted by two-phase Bligh-Dyer mixtures containing chloroform, methanol and water (2:2:1.8, v/v/v). The samples were vortexed to extract lipid A and then centrifuged at 2000 rpm for 20 min to separate. The lower phase containing lipid A was transferred to a round-bottom flask and dried through a rotary evaporator. The dried lipid A samples were solubilized in chloroform/methanol (2:1, v/v) and stored at −80 °C.

Negative ion Matrix Assisted Laser Desorption Ionization (MALDI)-TOF MS experiments were performed as described elsewhere [41]. Briefly, lipids were solubilized in 100 μL chloroform/methanol (2:1, v/v) and spotted (1 μL) directly onto the MALDI sample plate, followed by 1 μL of 100 mg/mL norharmane MALDI matrix dissolved in chloroform/methanol/water (3:1.5:0.25, v/v/v). All experiments were performed using a Bruker Autoflex Speed MALDI-TOF/TOF mass spectrometer (Bruker Daltonics Inc., Billerica, MA, USA). Each spectrum was an average of 500 shots and 50% laser power. ES Tuning Mix (Agilent, Palo Alto, CA, USA) was used as a calibration standard. Norharmane matrix was used throughout these studies.

### 2.6. Purification and Concentration Measurement of Lipopolysaccharide (LPS) from BL21 (DE3) and Its Mutants

LPS were purified from BL21 (DE3) and mutant strains by the phenol-water procedure with some modification [42]. Harvested bacteria (500 mg) were suspended with 15 mL of 10 mM Tris-Cl buffer (pH 8.0), containing 2% SDS, 4% 2-mercaptoethanol, and 2 mM MgCl_2_ in a centrifuge vessel. The suspension was placed in a 65 °C water bath until bacteria were solubilized. A total of 100 μg proteinase K was added in the suspension and the sample was incubated at 65 °C water bath for an hour, after which the sample was placed in a 37 °C water bath overnight. Two mL of 3 M sodium acetate was added to the proteinase K-digested cell suspension and then 40 mL of pre-chilled ethanol was added to the bacteria suspension and incubated at −20 °C overnight to form precipitate. The sample was centrifuged at 4000× *g* for 15 min and supernatant was decanted slowly; the precipitate was resuspended in 9 mL of distilled water. One mL of 3 M sodium acetate was added in the mixture before adding another 20 mL of chilled absolute ethanol, vortexing after each step. The suspension was placed at −20 °C overnight. After centrifugation, the precipitate was suspended by 9 mL 10 mM Tris-Cl (pH7.4), and then, the mixture sample was placed at 37 °C for 4 h with 50 μg DNase I and 12.5 μg RNase to digest residual contaminating nucleic acids. To remove all residual protein contaminants from LPS nuclease-treated mixture, the next step was a phenol extraction. The LPS mixture was placed in a 65 °C water bath for 30 min and an equal volume of 90% phenol that preheated to 65 °C was added. Subsequently, the mixture was placed at 65 °C for 15 min, cooled to 4 °C on ice and then centrifuged at 6000× *g* for 15 min. The aqueous top layer was collected in a new vessel and the phenol layer extracted with an equal volume of distilled water again. The mixture sample was heated to 65 °C again for 15 min and then placed in an ice-water environment to chill. After centrifugation, the top aqueous layer was added to the first aqueous extraction, all aqueous layers were dialyzed against multiple changes of distilled water over 2 days. After dialysis, the lipopolysaccharide was lyophilized and then dissolved in water to be stored at −80 °C until analyzed.

As the molecular weight of LPS samples from BL21 (DE3) and its derivatives are variable, and LPS molecule interacts with MD2-TLR4 to form a complex with a molecular ratio (1:1:1) to initiate the intracellular signaling, LPS samples were quantitated using the 3-deoxy-d-manno-octulosonic acid (Kdo) method according to published procedures [43], and the LPS were calculated and diluted to the appropriate concentration (Molar) for each experiment based on two Kdo moieties present in the LPS of the BL21 (DE3) and its derivatives.

### 2.7. LPS Stimulation of the Murine Macrophage Cell RAW264.7 and Human Monocyte Cell THP1

Murine macrophage cell line RAW264.7 and human monocyte cell THP1 were obtained from the cell bank of the Chinese Academy of Science (Shanghai, China). The cell lines were maintained at 37 °C with 5% CO_2_ in DMEM (Gibco BRL, Gaithersburg, MD, USA) containing 10% FBS (Hyclone, Logan, UT, USA) and in RPMI-1640 Medium (Gibco BRL, Gaithersburg, MD, USA) containing 10% FBS (Hyclone, Logan, UT, USA), respectively. For cell differentiation, THP-1 cells were treated with 10 mM phorbol 12-myristate 13-acetate (PMA) (Sigma-Aldrich, St. Louis, MO, USA) and incubated at 37 °C in 5% CO_2_ for 48 h. Cells were cultured to the concentration of 10^5^ per milliliter and seeded into 96-well plates with 200 μL medium each well. After 18 h, cells were stimulated with increasing concentration of LPS (10, 10^2^, 10^3^ and 10^4^ pM). After 24 h, culture supernatants were collected, centrifuged to pellet debris and stored at −80 °C for cytokine analysis. The concentrations of TNF-α and IL-12 were determined using the enzyme-linked immunosorbent assay kit (R & D Systems, Minneapolis, MN, USA), according to the manufacturer’s instruction.

One-way analysis of variance (ANOVA) was performed to determine the statistical significance of the differences between mean values for various experimental and control groups. Data were expressed as means ± one standard deviation, and the experiments were performed with three biological replicates. The means were compared using the least significant difference test. *p* < 0.05 was considered a significant difference and *p* < 0.01 was considered extremely significant difference. All data were analyzed with Statistical Product and Service Solutions (SPSS) Statistics 17.0 (Chicago, IL, USA).

## 3. Results

### 3.1. Construction of BL21 (DE3) ∆msbB28 ∆pagP38 Mutant and Its Derivatives

**Figure 1:**
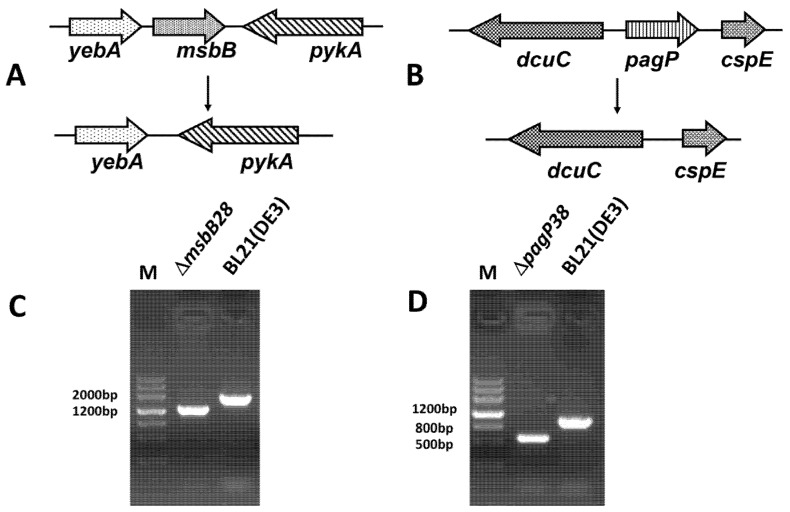
Construction of BL21 (DE3) mutant strain. (**A**) Map of deletion mutant resulting from knockout of *msbB* gene; (**B**) Map of deletion mutant resulting in knockout of *pagP* gene; (**C**) The identification result of ∆*msbB28* mutant, the result shows that *msbB* gene was deleted; (**D**) The identification result of ∆*pagP38* mutant, the result shows that *pagP* gene was deleted.

Two suicide plasmids pQK001 and pQK002 were constructed to facilitate deletion of *msbB* and *pagP* gene in the BL21 (DE3), respectively, and the gene deletions were performed by homologous recombination via SacB-based counter-selection in allelic-exchange experiments [37]. Figure 1 illustrates the chromosomal structures of ∆*msbB*28 and ∆*pagP*38 in BL21 (DE3), and their PCR product size when amplified by flanking primers. The intermediate single ∆*msbB*28 or ∆*pagP*38 mutants were further confirmed by DNA sequencing. ∆*pagP*38 was introduced into intermediate mutant ∆*msbB*28 to yield the mutant S001 (∆*msbB*28 ∆*pagP*38), which was hypothesized to result in production of penta-acylated lipid A in this progeny mutant strain (Table 2).

**Figure 2:**
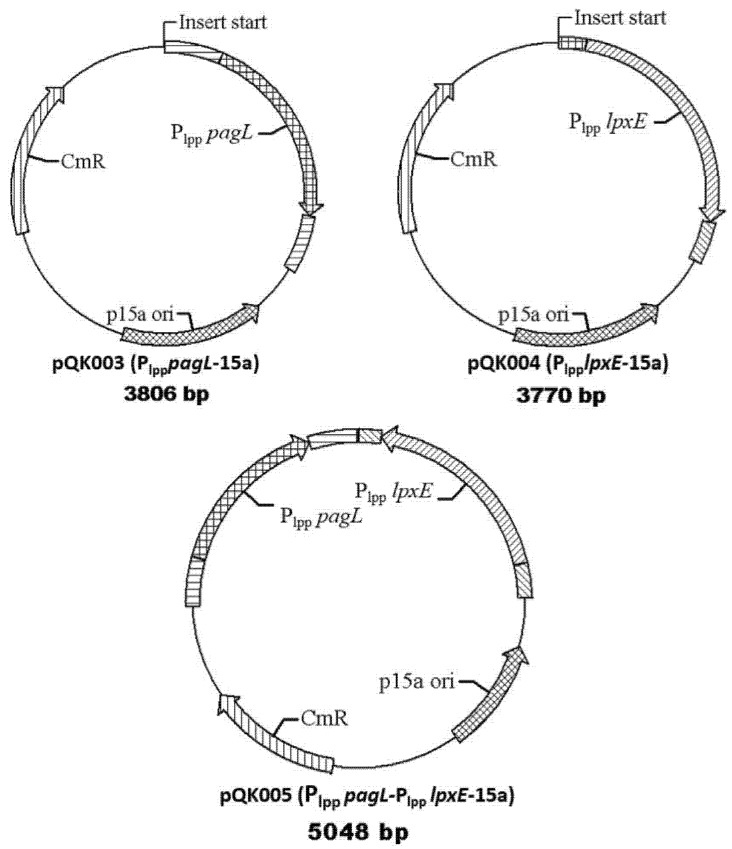
Plasmid maps used in this study. Maps of three expression plasmids constructed in this study. Plasmid pQK003 was constructed for expressing *pagL* to decrease the fatty acyl chain number. Plasmid pQK004 was constructed for expressing *lpxE* to remove 1-phosphate group in lipid A. Plasmid pQK005 was constructed for expressing *pagL* and *lpxE* to confer both of these functions.

The other three plasmids were constructed for overexpressing *pagL* (pQK003), *lpxE* (pQK004), and both *pagI* and *IpxE* (pQK005) under constitutive transcriptional control of the strong *E*. *coli* promoter P_lpp_ (Figure 2). These plasmids replicate with low copy number in *E. coli* (origin of replication p15a) and encode a Cm resistance marker to facilitate use in other bacterial strains. Plasmids pQK003, pQK004 and pQK005 were transformed separately to S001 to yield the strains S002, S003 and S004, respectively (Table 2).

### 3.2. Structure Analysis of Lipid A Extracted From BL21(DE3) and Its Derivatives

**Figure 3:**
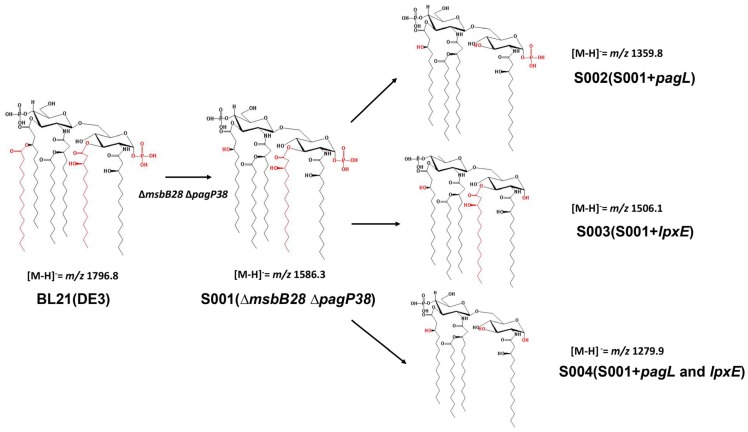
The schematic structure of lipid A. The number of phosphate and fatty acyl chain of lipid A is indicated and the spectrum number *m*/*z* of lipid A structure is shown. The deletion of *msbB* and overexpression of *pagL* could change the number of fatty acyl chains of lipid A. Expression of *lpxE* in *E. coli* could remove the phosphate group of lipid A. Both structural modifications therefore change lipid A to tetra-acylated monophosphoryl lipid A.

**Figure 4:**
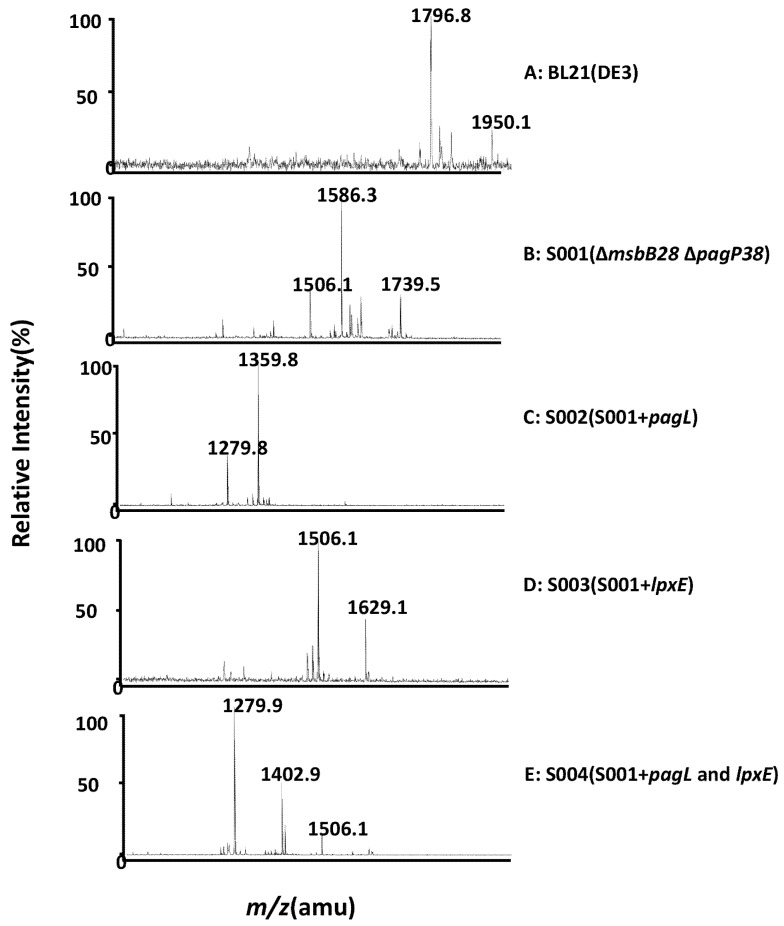
MALDI-TOF MS analysis of lipid A isolated from BL21 (DE3) mutants. Lipid A samples were dissolved in chloroform/methanol (4:1, v/v) and subjected to MALDI-TOF MS in the negative ion mode. The parental strain BL21 (DE3) makes a hexa-acylated lipid A as indicated by the [M − H]^−^ peak at *m*/*z* 1796.8. The mutant strain S001 makes a penta-acylated lipid A as indicated by the [M − H]^−^ peak at *m*/*z* 1586.3. Removal of one phosphate group shifts the lipid A [M − H]^−^ peak by *m*/*z* −70. Removal of a fatty aycl chain shifts the MS peak by *m*/*z* −226.5.

Lipid A were extracted from BL21(DE3) and its derivatives by the Bligh-Dyer method [44], and analyzed by MALDI-TOF-MS (Figure 3 and Figure 4). The lipid A isolated from parent strain BL21(DE3) contains a predominant hexa-acylated lipid A (*m*/*z* 1796.2 in Figure 4) and a minor peak of hexa-acylated lipid A decorated with a 4-amino-4-deoxy-l-arabinose (l-Ara4N) residue (*m*/*z* 1950.1), which is consistent with other reports [45]. S001 (∆*msbB28* ∆*pagP38*) yielded a major peak at *m*/*z* 1586.3 in the spectrum, resulting from failure of myristic acid chain addition to the 3′-position of lipid A due to the *msbB* deletion (Figure 3 and Figure 4B), and there was the second major peak at *m*/*z* 1739.5, which was a penta-acylated lipid A modified with l-Ara4N. As expected, PagL expression in the strain S002 lead to a major peak of 3-*O*-deacylated lipid A at *m*/*z* 1359.8 in the spectrum, which is tetra-acylated lipid A (Figure 3 and Figure 4C), and LpxE expression resulted in production of mono-phosphorylpenta-acylated lipid A (*m*/*z* 1506.1) and a minor peak at *m*/*z* 1629.1 which is a mono-phosphorylpenta-acylated lipid A derivative of a phosphoethanolamine (pEtN) addition to the 4′-phosphate position in the strain S003 (Figure 4D); in the spectrum of lipid A isolated from the strain S004 with both PagL and LpxE expression, there are two major peaks at *m*/*z* 1279.9 and *m*/*z* 1402.9 and a minor peak at *m*/*z* 1506.1; the peak at *m*/*z* 1279.9 is a dephosphorylated tetra-acylated lipid A at the 1-position, and a pEtN addition to dephosphorylated tetra-acylated lipid A at the 4′-position lead to the peak at *m*/*z* 1402.9, and the peak at *m*/*z* 1506.1 is a mono-phosphorylpenta-acylated lipid A, indicating that PagL is not fully functional in the strain S004.

There are some other minor peaks in the lipid A mass spectrum isolated from the BL21(DE3) strain and its derivatives, such as peak at *m/z* 1506.1 in the S001 and peak at 1279.8 in strain S002, which might arise by loss of a phosphate due to the harsh hydrolysis of cleavage of the lipid A from the core-saccharide, or may result from the small molecules addition to the lipid A when performing lipid A analysis by mass spectrum [27].

### 3.3. Comparison of the Endotoxic Activities of LPS Isolated from BL21 (DE3) and Its Derivatives

The effect of modified lipid A on cytokine stimulation in tissue culture was determined by measuring TNF-α in macrophage cell line RAW264.7 originated from mouse and THP1 cells derived from human, representing two distinct TLR4 receptors [46,47]. LPS was purified from BL21 (DE3) and its derivatives by hot-phenol method and repurified with deoxycholate-phenol to eliminate lipoproteins, ensuring no other known pathogen-associated molecular patterns (PAMPs) were present in the LPS samples when measuring endotoxic activity [48].

**Figure 5:**
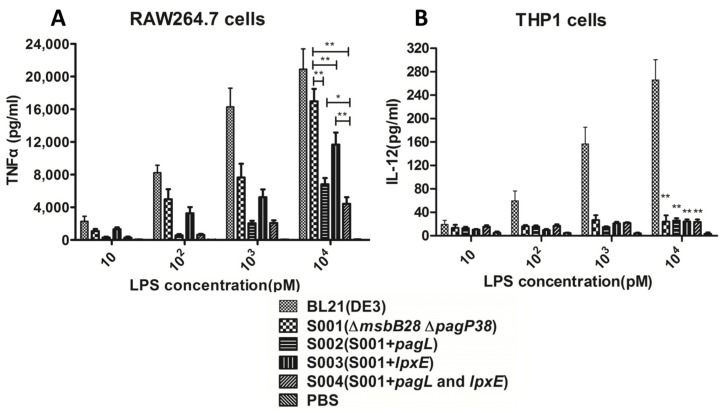
Cytokine concentrations in culture supernatants of RAW264.7 cells and THP1 cells stimulated by different concentrations of lipopolysaccharide (LPS) samples from *E. coli* BL21 (DE3) and mutant strains. Supernatant samples from cells were collected after 24 h stimulation with LPS and assayed for the production of inflammatory factor. (**A**) TNF-α released into the culture supernatant of RAW264.7 cells stimulated for 24 h with different concentration of LPS; (**B**) IL-12 released into the culture supernatant of THP1 cells stimulated for 24 h with different concentration of LPS. One-way analysis of variance was used to evaluate differences in cytokine concentrations, significant differences between the parental strain groups and other mutant strain groups stimulated by LPS were shown. * *p* < 0.05; ** *p* < 0.01. The experiment was performed in triplicate, and data are shown as means ± standard deviation, with PBS group as negative control.

The TNF-α in the RAW264.7 and IL-12 in the THP1 were measured after 24 h stimulation by LPS of various concentrations from 10 pmol/L to 10^4^ pmol/L (Figure 5 and Supplementary Table S1). The levels of TNF-α in the RAW264.7 were proportional to the concentration of LPS isolated from BL21 (DE3) and its derivatives, and all types of LPS including tetra-acylated and dephosphorylated lipid A acts as agonists to activate the mouse TLR4-pathway.

At all concentrations, the level of TNF-α in the RAW264.7 stimulated by LPS from BL21 (DE3) was significantly higher than that stimulated by other modified LPS, indicating that hexa-acylated lipid A from the wild-type *E*. *coli* strain is the most endotoxic form of these strains in activating the TLR4-pathway, with the TNF-α levels from 2300 pg/mL to 20,900 pg/mL induced by BL21 (DE3) LPS at 10 pmol/L to 10^4^ pmol/L. The LPS purified from S001 (∆*msbB28* ∆*pagP38*) induced significantly higher levels of TNF-α than did other S001 expressing *pagL*, or *lpxE* or both, and LPS purified from S004 (*pagL* and *lpxE*) induced the significantly lower levels of the pro-inflammatory cytokines TNF-α than did the S002 (*pagL*) and S003 (*lpxE*), but still induced 4427 pg/mL TNF-α in the mouse macrophage cell line RAW264.7 at 10^4^ pmol/L, indicating that monophosphoryl tetra-acylated lipid A still acts as an agonist when interacting with mouse TLR4.

The levels of IL-12 released by human THP1 cells were proportional to the concentration of LPS isolated from the parent strain BL21 (DE3) and its derivatives. The IL-12 levels from 19.4 pg/mL to 266 pg/mL were induced by BL21 (DE3) LPS at 10 pmol/L to 10^4^ pmol/L. The LPS from other BL21 (DE3) derivatives showed the ability to stimulate lower levels of the production of IL-12 in the THP1 cell line, but the levels of IL-12 did not increase proportionally to each LPS concentration. No difference of the levels of IL-12 in the THP1 cell line was observed when stimulation of the LPS from the S001 (∆*msbB*28 ∆*pagP*38) and the S001 expressing *pagL*, or *lpxE* or both, indicating that penta-acylated lipid A from S001 (∆*msbB*28 ∆*pagP*38) containing five fatty acyl chain and further modified lipid A behaves as an antagonist that does not activate human TLR4 receptor [16,17].

### 3.4. Lipid A Modification Did Not Change the Growth Kinetics of the BL21 and Its Derivatives and They Retain Ability to Produce Recombinant Proteins

**Figure 6:**
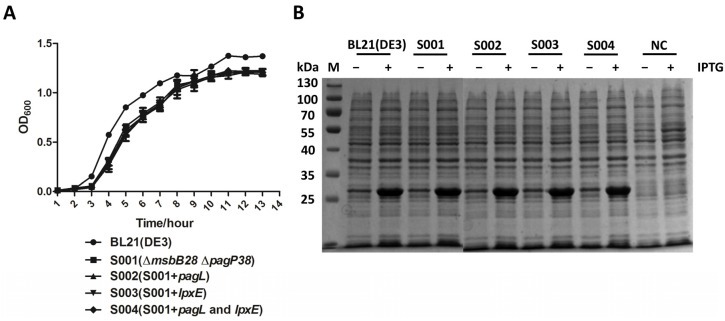
Biological characteristics analysis of BL21 (DE3) mutant strains. (**A**) Comparison of growth of BL21 (DE3) mutants, the experiment was performed in triplicate, and data were shown as means ± standard deviation; (**B**) Expression of PhoP in BL21 (DE3) and mutant strains. The recombinant proteins and strain used for expression are indicated in this figure. M: Protein ladder; 1: BL21 (DE3); 2: S001 (∆*msbB28* ∆*pagP38*); 3: S002 (S001 + *pagL*); 4: S003 (S001 + *lpxE*); 5: S004 (S001 + *pagL* and *lpxE*); NC: Negative control, BL21 (DE3) containing the control plasmid.

We measured the growth curves of the BL21 (DE3) and its derivatives to investigate whether the lipid A modification influences the bacterial growth as it is an essential feature to be considered if the bacterial strains would be used as hosts to express heterologous proteins. The modification of lipid A had no significant effect on growth rates of the BL21 (DE3) derivatives. The growth rates of all the derivative strains were similar to that of the parent strain BL21 (DE3) (Figure 6A); therefore, these BL21 derivative strains with low entodoxic lipid A were suitable for application of expressing recombinant proteins.

We then investigated their abilities to produce recombinant proteins in the BL21 (DE3) and its derivatives (Figure 6B). The comparison was performed using the protein PhoP, a global regulatory protein from *Pasteurella multocida* 8081, for their variable levels of expression in BL21 (DE3). No significant differences were observed between the four mutant strains and the parent BL21 strain in the levels of recombinant protein production after induction by IPTG, indicating that the lipid A modification did not compromise the ability to produce the recombinant protein.

## 4. Discussion

BL21 (DE3) and its derivatives are widely used for protein expression and purification for many purposes. One concern when using these proteins for clinical purposes is LPS contamination, leading to unacceptable reactogenicity. Therefore, the endotoxic LPS has to be removed from recombinant proteins before their use for therapeutic purpose. Previous studies demonstrated that a non-myristoylated (penta-acylated) LPS resulting from inactivation of *msbB* in *E*. *coli* and *Salmonella* exhibited markedly less ability to induce pro-inflammatory TNF-α in human monocytes [14,39,49]. To minimize the endotoxic activity of the LPS, lipid A structure was further modified in this study by removal of 3-*O*-acylation chain and phosphate group in the BL21 (DE3) *msbB* mutant using plasmids to express *pagL* and *lpxE* genes. We also included *pagP* deletion in the BL21 (DE3) to avoid the potential addition of palmitate chain to the lipid A [20,50]. Initially, we planned to inactivate *htrB* gene in the *msbB* mutant, which would result in production of tetra-acylated lipid A, but the double mutant exhibited abnormal growth in the LB media (data not shown). We then opted to select the *pagL* to selectively remove 3-*O*-acylation of lipid A [51,52].

The MS data demonstrated that PagL and LpxE expression from plasmid could lead to complete production of tetra-acylated lipid A and mono-phosphorylated penta-acylated lipid A, and both PagL and LpxE expression in one plasmid resulted in production of mono-phosphorylated tetra-acylated lipid A while minor decorations such as pEtN or Ara4N were present in the lipid A (Figure 2 and Figure 3) [27]. No data were currently available to demonstrate whether these minor modifications on the phosphate position of lipid A impact its interaction with the TLR4 receptor. Therefore, in this study we still retained *arnT* and *eptA* genes, which are responsible for pEtN or Ara4N addition to the lipid A, in the BL21 (DE3) chromosome [53,54].

Toll-like receptors (TLRs) have an essential function in innate and adaptive immunity by responding to microbial components, and they are expressed mainly on antigen-presenting cells such as monocytes-macrophages and dendritic cells [7]. TLR4, as the receptor for the Gram-negative bacterial LPS, can mediate the signal of macrophages for cytokine production such as IL-6, IL12 and TNF-α [15,16,28]. We therefore chose the macrophage cell line RAW264.7, originated from mouse, and THP1 cells derived from human to investigate the effect of LPS modification to TLR4 activity and evaluate the endotoxic activity of that lipid A in mammalian and human cells.

Previous reports show *msbB* deletions in BL21 (DE3) and that other bacteria reduce the capacity of LPS from these *msbB* mutants to activate human-derived immature monocyte-derived DC by more than two orders of magnitude [16,55]. Similarly, we have shown that LPS isolated from S001 (∆*msbB28* ∆*pagP38*) also has strongly reduced its capacity to stimulate IL12 production by human THP1, but still retained its capacity to trigger TNF-α in RAW264.7 cells derived from mouse (Figure 5). Removal of the 3-*O*-acylation chain or phosphate group in the penta-acylated LPS produced by S001 (∆*msbB28* ∆*pagP38*) resulted in greatly decreased capacity to trigger TNF-a production in RAW264.7, and the former significantly reduced the potency in triggering TNF-α production in RAW264.7 compared with effect of removal of phosphate group (Figure 5), indicating that alteration of fatty acid number of lipid A would be a more efficient approach to decrease its endotoxic activity than removal of phosphate group [27,56]. While LPS isolated from the S003 (*pagL* and *lpxE*) greatly reduced its ability to induce TNF-α in the RAW264.7 cell, this mono-phosphorated tetra-acylated LPS induces pro-inflammatory response in the mouse cell. Further modifications of the lipid A of the BL21 (DE3) to minimize its endotoxic activity by other approaches, such as inactivation of *htrB* and removal of the second phosphate group by *lpxF*, can remove the 4′-phosphate group [57,58].

BL21 (DE3) and its derivatives were tested for expression of recombinant proteins from pET32a plasmids encoding by *phoP* and other genes from *Pasteurella multocida* 8081 and *Salmonella typhimurium*. While lipid A modification has minor effects on bacterial growth, the levels of expression were indistinguishable from the original BL21 (DE3) strain (Figure 6 and data not shown), which was consistent with the previous reports [16]. This also provided us with a basis to genetically engineer the BL21 (DE3) to further reduce its endotoxic activity for a future study.

We chose plasmid vectors to express *pagL* and *lpxE* because it is convenient for transferring plasmids to other bacterial strains for other purposes; for example, plasmid pQK004 carrying *lpxE* or pQK005 carrying *lpxE* and *pagL* can be used for production of mono-phosphoryl lipid A, which can be used as vaccine adjuvants [26,27]. While insertion of these genes into the bacterial chromosome could avoid utilization of selection antibiotics, these chromosome-integrated genes are not always expressed enough for fully modifying the lipid A structure [27,37]; moreover, we can take advantage of balanced-lethal host-vector systems to stably maintain plasmids without use of antibiotic as selective pressures in BL21 (DE3) or other bacterial strains [59,60]. Therefore, plasmid vector is still an ideal option to express heterologous genes in *E. coli.*

## 5. Conclusions

In all, in this study we have succeeded in constructing a BL21 ∆*msbB* mutant and its derivatives, and demonstrated that these progeny strains contained modified forms of lipid A with markedly decreased capacity to induce the inflammatory response in human and mouse cells. These mutant strains may be used for expressing therapeutic recombinant proteins or targeted drugs without elimination of LPS.

## Supplementary Files

Supplementary File 1
