# Supplementary material for: Construction of Escherichia coli Mutant with Decreased Endotoxic Activity by Modifying Lipid A Structure
Source: Mar Drugs. 2015 May 27;13(6):3388–406. doi: 10.3390/md13063388 (PMC4483635; doi:10.3390/md13063388)
Supplement: Supplementary File 1 [file marinedrugs-13-03388-s001.pdf]

Supplementary Information

Table S1. Cytokine concentrations in culture supernatants of RAW264.7 cells and THP1 cells stimulated by different concentrations of lipopolysaccharide (LPS) sample from *E. coli* BL21 (DE3) and mutant strains.

**Cells**	**LPS Concentration (pM)**	**BL21 (DE3)**	**S001 (Δ*msbB28* Δ*pagP38*)**	**S002 (S001 + *pagL*)**	**S003 (S001 + *lpxE*)**	**S004 (S001 + *pagL* and *lpxE*)**	**PBS**
TNF-α from RAW264.7 cells (pg/mL)	10	2300 ± 613	1075 ± 267	296 ± 97	1319 ± 219	274 ± 142	53 ± 10
	10^2	8226 ± 923	4492 ± 1219	568 ± 147	3266 ± 763	627 ± 118	21 ± 7
	10^3	16,293 ± 2280	7648 ± 1684	2051 ± 297	5236 ± 953	2080 ± 335	44 ± 14
	10^4	20,904 ± 2480	16,972 ± 1514	6825 ± 767 †	11,677 ± 1470 †	4427 ± 821 *,†	65 ± 15
IL-12 from THP 1 cells (pg/mL)	10	19.4 ± 6.9	13.5 ± 5.2	12.9 ± 2.4	10.6 ± 0.9	15.4 ± 2.7	5.3 ± 2.1
	10^2	59.6 ± 16.7	16.1 ± 2.1	15.5 ± 2.1	9.6 ± 2.0	17.0 ± 2.6	4.6 ± 1.0
	10^3	156.6 ± 28.7	26.7 ± 8.2	14.7 ± 1.3	21.3 ± 2.5	21.9 ± 1.0	4.0 ± 1.5
	10^4	266.0 ± 34.4	24.2 ± 10.6 **	26.0 ± 4.1 **	24.4 ± 3.4 **	23.6 ± 4.2 **	3.2 ± 2.6

One-way analysis of variance was used to evaluate differences in cytokine concentrations. Significant differences between the parental strain groups and other mutant strain groups stimulated by LPS were shown. † $p < 0.01$ compared with S001; * $p < 0.05$ compared with S002; ** $p < 0.01$ compared with parental strain BL21 (DE3). The experiment were performed in triplicate, and data were shown as means ± standard deviation, PBS group was negative control.

© 2015 by the authors; licensee MDPI, Basel, Switzerland. This article is an open access article distributed under the terms and conditions of the Creative Commons Attribution license (http://creativecommons.org/licenses/by/4.0/).
